# Repeatability of Health and Welfare Traits and Correlation with Performance Traits in Dairy Goats Reared under Low-Input Farming Systems

**DOI:** 10.3390/vetsci9060289

**Published:** 2022-06-11

**Authors:** Sotiria Vouraki, Athanasios I. Gelasakis, Vasileia Fotiadou, Georgios Banos, Georgios Arsenos

**Affiliations:** 1 Laboratory of Animal Husbandry, School of Veterinary Medicine, Faculty of Health Sciences, Aristotle University, 54124 Thessaloniki, Greece; vafot@vet.auth.gr (V.F.); or banos@vet.auth.gr (G.B.); arsenosg@vet.auth.gr (G.A.); 2 Department of Animal Science, School of Animal Biosciences, Agricultural University of Athens, 11855 Athens, Greece; gelasakis@aua.gr; 3 Department of Animal and Veterinary Sciences, Scotland’s Rural College (SRUC), Easter Bush, Midlothian EH25 9RG, UK

**Keywords:** repeatability, correlation, dairy goats, health, welfare, performance, low-input farming

## Abstract

The objectives of the study were to estimate the repeatability of health and welfare traits and investigate their association with performance in three breeds of dairy goats reared under low-input farming systems in Greece. A total of 1210 goats of Eghoria (*n* = 418), Skopelos (*n* = 429), and Damascus (*n* = 363) breeds were assessed. Udder health, parasitic resistance, welfare, milk yield and quality, and body condition score were recorded monthly for two milking periods. Udder health records included somatic cell count (SCC) and total viable count (TVC). Based on combinations of SCC and TVC and thresholds set at >10^6^ cells/mL and >2 × 10^4^ cfu/mL, respectively, additional udder health phenotypes were defined. Parasitism included myiasis, tick infestation, gastrointestinal nematode (GIN) and cestode faecal egg count (FEC), and lungworm faecal larval count (FLC). Infection with each of the endoparasites was defined based on FEC/FLC. Welfare assessment parameters included the presence of ear and horn injuries, ocular and nasal discharge, body and udder abscesses, injury and lesions on the skin of different regions, diarrhoea, hernias, overgrown hooves, arthritis, lameness, and udder asymmetry. Trait repeatability and animal correlations were estimated. Significant (*p* < 0.05) repeatability was reported for all udder health and most welfare traits in all breeds, GIN and cestode FEC, and GIN and lungworm infection in Eghoria, and myiasis in Skopelos. Correlations of health and most of welfare traits with performance were non-significant or favourable. Overall, results demonstrate potential to improve health and welfare of the studied breeds without compromising performance.

## 1. Introduction

Dairy goat farming is a significant animal husbandry activity with an important socioeconomic and environmental role in disadvantaged areas [1]. Given the ability of goats, especially of indigenous breeds, to utilise diverse vegetation in mountainous and marginal regions, they are traditionally reared under low-input pastoral farming systems [2,3]. The notion is that the sustainability of such systems depends on animal productivity [2]. However, the latter is defined by the status of animal health and welfare that is directly associated with environmental conditions, housing conditions, and herd health management [4,5].

Grazing at natural pastures favours internal and external parasitism, which affects goat health and welfare status [4,5,6]. Moreover, traditional goat shelters are frequently characterised by high stocking density, inadequate bedding, and poor hygiene, which predispose animals to bacterial infections, particularly subclinical mastitis, with a cumulative incidence of 24–32% recently reported in low-input pastoral farming systems [7]. Additionally, poor husbandry conditions and hygiene status can lead to welfare issues including injuries, abscesses and skin lesions, udder problems, and limb disorders [4,5,8].

Preventive and treatment measures to mitigate the occurrence and consequences of the above health and welfare issues can be challenging. Specifically, the repeated use of anthelmintics for controlling gastrointestinal nematodes (GIN) has led to increasing anthelmintic resistance [9,10,11]. At the same time, chemical control of ectoparasites with zero withdrawal time is perceived as rather expensive by the farmers [12]. Moreover, preventive strategies against subclinical mastitis can be cumbersome and expensive, especially for large-size herds [13]. Genetic selection for disease-resistant animals is a promising complementary approach to the above measures. Previous studies have shown the possibility for selective breeding towards enhanced udder health [14,15,16,17] and GIN resistance [18,19,20] in goats.

Genetic selection, however, is often limited by the lack of reliable pedigree records in low-input pastoral dairy goat farms [5,20]. Nevertheless, investigating between-animal (co)variation of performance, health, and welfare traits, even without pedigree data, could still inform decision-making for improved management and selection practices at the animal level towards enhanced overall productivity. Such an investigation is feasible by estimating the repeatability of health and welfare traits and the animal correlations with performance traits. This could help identify any potential for improving goat health and welfare status without compromising performance.

In goats, relevant studies are scarce. Previous research has investigated the repeatability of somatic cell count (SCC) [14,16,17,21]. To the best of our knowledge, there are no references regarding other indicators of udder health status such as total viable count (TVC). Moreover, studies on goat parasitism have reported trait repeatability for GIN based on faecal egg count (FEC) [9,10,22,23], but there has been no research on resistance to other endoparasites or ectoparasites. There have been no relevant studies on welfare-related traits and their association with goat performance.

The objectives of the present study were to (i) estimate the repeatability of a wide range of health and welfare traits measured on goats of three breeds reared under low-input pastoral farming systems in Greece, and (ii) investigate their association with milk production and body condition score (BCS).

## 2. Materials and Methods

### 2.1. Farms and Animals

**Figure 1:**
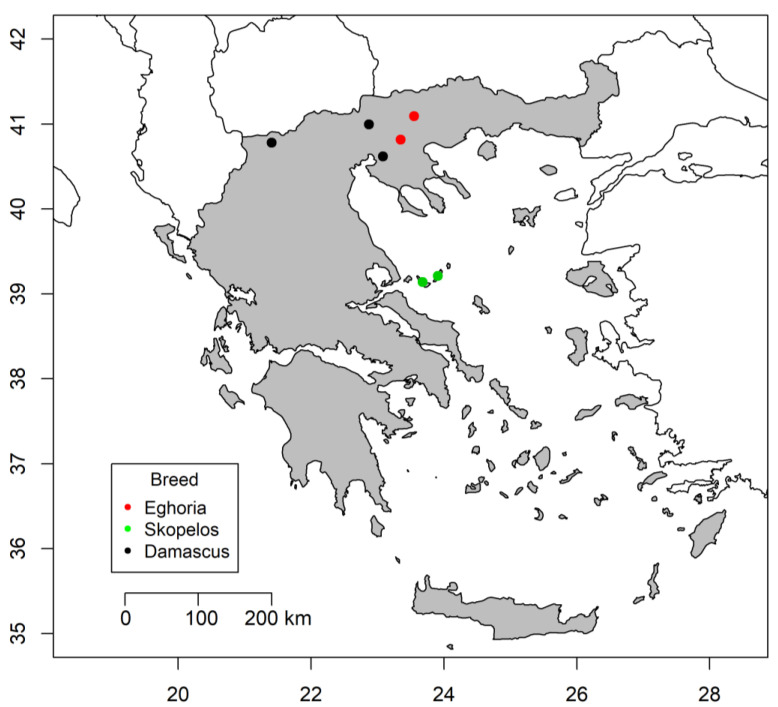
Map of Greece illustrating the regions in which the studied goat herds were located.

A total of 1210 dairy goats of two indigenous Greek breeds (Eghoria, *n* = 418 and Skopelos, *n* = 429) and one imported breed (Damascus, *n* = 363) were used. Animals were randomly selected from seven farms (two with Eghoria, two with Skopelos, and three with Damascus goats) located in northern and central Greece (Figure 1). All herds were selected as representatives of the studied breeds and the low-input pastoral farming system, the typical goat production system in Greece [24]. This system is characterised by grazing shrubland and woodland areas throughout the year, traditional management practices, and random mating of animals.

### 2.2. Phenotypic Data and Recording Protocols

Data collected during FP7-SOLID project (Sustainable Organic Low Input Dairying, 266367, 2011–2015) were used for this study. The available dataset included individual monthly records for two consecutive milking periods (corresponding to kiddings between November 2011 and January 2012, and November 2012 and January 2013; five records per milking period) of animal health, welfare, and production traits.

Specifically, a standard protocol for welfare trait assessment was developed based on Anzuino et al. [25]. Prior to milking, each goat was restrained and assessed through observation and palpation of the head, body, udder, and limbs for ear and horn injuries, head skin lesions (hair loss and/or dermatitis), ocular and nasal discharge, body abscesses and injuries, diarrhoea, hernia, overgrown claws, arthritis, udder asymmetry, udder abscess, and udder skin lesions. Moreover, a locomotion score was assigned using the 5-point scale (1: normal gait; 2: no obvious lameness when standing, occasional limping when walking; 3: lifting foot while standing and moderate lameness when walking; 4: shifting stance and severe lameness when walking; 5: unwilling to bear weight on one foot when standing or walking) of Ley et al. [26]. All the above traits were consistently assessed by the same veterinarian. A detailed description of the welfare traits assessed is available in the study by Gelasakis et al. [5].

All goats were also thoroughly examined individually for the presence of hard ticks throughout the body and warbles beneath the skin on the back caused by *Przhevalskiana silenus* larvae. Additionally, a faecal sample was collected to measure FEC of GIN and cestodes, and faecal larval counts (FLC) of lungworms using the modified McMaster technique [27].

The BCS of each goat was assessed by palpation of the dorsal lumbar region and recorded on the 5-point scale (1: emaciated to 5: obese, in 0.5-point increments) of Russel et al. [28]. Subsequently, a milk sample was collected aseptically from both udder halves to be tested for TVC. Each goat was then hand-milked into a bucket, the milk was weighed, and a milk sample was collected from the milking bucket to assess SCC and milk composition (fat, protein lactose, and solids-non-fat (SNF) content). Sampling and analyses of milk samples have been described in detail in previous studies [7,29,30].

### 2.3. Phenotypic Data Handling

The official AT method of the International Committee of Animal Recording [31] was used to calculate individual daily milk yield of the studied goats by doubling the recorded yield of the morning or evening milking. Daily milk fat, protein, lactose, and SNF yields were also calculated from milk and the corresponding content records.

Moreover, some additional health and welfare phenotypes were defined based on the measurements described above. Specifically, a subclinical mastitis index (SMI) was derived using the following formula:

(1) $$\mathrm{SMI}=0.6\frac{\mathrm{SCC}\;-\;\mathrm{Mean}}{\mathrm{SD}}\;+\;0.4\frac{\mathrm{TVC}\;-\;\mathrm{Mean}}{\mathrm{SD}}$$

where Mean and SD correspond to the mean and standard deviation, respectively, of each trait (SCC and TVC) within each studied breed.

Thresholds were also set for SCC and TVC at >10^6^ cells/mL and >2 × 10^4^ cfu/mL [7], respectively, and used to define three additional udder health phenotypes: (i) UHP1, scored as 0 or 1 if at least one of the traits was below or both were above the thresholds, respectively, (ii) UHP2, scored as 0–2 if both traits were below, only one was above, or both exceeded the thresholds, and (iii) UHP3 scored as 0–3 if both traits were below, only TVC was above, only SCC was above, or both were above the thresholds.

Furthermore, FEC and FLC records were used to determine the presence or absence of infection with GIN and cestodes, and lungworms, respectively. In all cases, records of ≥50 FEC or FLC were indicative of infection [32]. We also defined an overall endoparasite infection index, where at least one of these parasites was detected. Moreover, the presence of at least 10 ticks attached to the body of goats was considered as tick infestation [6,33]. A myiasis case was defined by the presence of at least two warbles beneath the skin on the back of the animal.

Regarding welfare traits, the locomotion score assigned to each animal was used to define the presence or absence of lameness. In addition, measurements on each body part were used to define the presence or absence of head, body, limb and udder problems, and skin lesions and injuries.

Descriptive statistics of all animal traits considered in the study are shown in Table 1, Table 2, Table 3 and Table 4. The final dataset used for the analyses is presented in Dataset S1.

### 2.4. Data Analysis

Milk production traits SCC and TVC were logarithmically transformed to ensure normality of distributions. FEC and FLC records, which were also significantly skewed, were transformed using Tukey’s Ladder of Powers with R package “rcompanion”.

Preliminary analyses were performed to identify environmental factors with statistically significant (*p* < 0.05) effects on the studied traits. The effects of farm, period of kidding, age at kidding, days from kidding, and interactions between them were tested.

(Co)variance components of health and welfare phenotypes with milk production traits and BCS were estimated within breed in a series of bivariate statistical analyses implemented with Bayesian Markov Chain Monte Carlo methods and the R software package “MCMCglmm” [34]. All analyses were based on the following model:

(2) $$Y_{\mathrm{ijmn}}{=\mu +F}_{i}P_{j}{+b1*A+b2*D+A}_{m}{+e}_{\mathrm{ijmn}}$$

where Y_ijmn_ = studied trait (n^th^ measurement on animal m); μ = overall population mean; F_i_P_j_ = fixed effect of the interaction between farm (I = two levels for Skopelos and Eghoria and three for Damascus) and period of kidding (j =two levels); b1 = regression coefficient on age at kidding A (months); b2 = regression coefficient on days from kidding D; A_m_ = random effect of the animal m; e_ijmn_ = random residual effect.

A probit link function for binomial distribution was fitted to model 2 for the analyses of binary traits.

Weekly informative priors were used for the random animal and residual effects, whereas for the fixed effects the default normal prior distribution with null mean and a large variance (10^10^ was used. For binary traits, residual variance was fixed to one [34]. For each bivariate model, three chains of 13,000 to 2,300,000 iterations with a burn-in period of 3000 iterations and a thinning interval of 10 to 2000 samples were used (depending on each model’s convergence and autocorrelation diagnostics). Convergence of the models was tested with visual inspection of estimate plots and the Gelman and Rubin’s convergence diagnostic, where values above 1 indicate lack of convergence [35]. Moreover, autocorrelation across chains was tested for all lag values greater than zero (values below 0.1 were considered acceptable).

Repeatability and animal correlations between studied traits were estimated from the posterior means of corresponding variance and covariance values after convergence. In the case of binary traits, results were expressed on the latent (liability) scale. Repeatability estimates of all studied traits were derived as the mean of estimates from multiple bivariate analyses.

## 3. Results

### 3.1. Trait Repeatability Estimates

Estimates of repeatability for health and welfare traits within breed (Eghoria, Skopelos, and Damascus) are shown in Table 5 and Table 6, respectively; for comparison, estimates of milk production traits and BCS are listed in Supplement Table S1. Statistically significant (*p* < 0.05) estimates were derived for all udder health traits ranging from 0.08 to 0.50, 0.09 to 0.59, and 0.08 to 0.43 in Eghoria, Skopelos, and Damascus goats, respectively. In all breeds, the most repeatable udder health trait was UHP1 (Table 5). Of the parasitism traits, significant repeatability estimates were found for GIN and cestode FEC, and GIN, lungworm and overall endoparasite infection in Eghoria goats (0.09–0.32) and for cestode infection (0.27) and myiasis (0.34) in Skopelos goats (Table 5). Regarding welfare, significant repeatability estimates were found for all traits except for ocular discharge, diarrhoea, injury and hernia in all breeds, lameness and arthritis in Eghoria and Skopelos goats, and nasal discharge in Skopelos goats. Significant repeatability estimates for welfare traits were 0.16–0.99, 0.19–0.97, and 0.20–0.98 in Eghoria, Skopelos, and Damascus goats, respectively; the most repeatable traits were ear, horn, and total injuries (Table 6). All studied milk production traits were significantly repeatable with estimates ranging from 0.26 to 0.41, 0.35 to 0.47, and 0.21 to 0.52 in Eghoria, Skopelos, and Damascus goats, respectively (Supplementary Table S1). Significant repeatability estimates were also found for BCS in all three breeds (0.31–0.47; Supplement Table S1).

### 3.2. Animal Correlations of Health and Welfare Traits with Milk Production Traits and Body Condition Score

Statistically significant animal correlations of health and welfare traits with milk production traits and BCS within breed are shown in Table 7 and Table 8, respectively. All animal correlations estimated are presented in detail in Supplement Tables S2–S4. Notably, a negative sign in the correlation is in the desirable direction signifying reduced incidence of health and welfare issues as performance improves. Significant negative correlations were reported between most udder health and milk production traits in all breeds (Table 7). Moreover, GIN and endoparasite infections were also negatively correlated with milk yield, lactose yield, and SNF yield in Eghoria goats (Table 7). Regarding welfare traits, negative correlations were reported between head skin lesions and protein yield, and total skin lesions and fat yield in Eghoria goats, and between udder asymmetry and all milk production traits in Damascus goats. In Damascus goats, arthritis and limb problems were also negatively correlated with BCS (Table 8). In contrast, udder abscess was positively correlated with protein yield in Eghoria goats and with most milk production traits in Skopelos goats (Table 8). In the latter breed, positive correlations were also identified between udder problems and protein yield, and of overgrown claws and limb problems with all milk production traits (Table 8).

## 4. Discussion

This study investigated the repeatability of a wide range of animal health and welfare traits and their association with milk production and BCS in three dairy goat breeds reared under low-input pastoral farming systems in Greece. Overall, significant between-animal variation was revealed for udder health, resistance to parasitism, and most studied welfare traits in Eghoria, Skopelos, and Damascus goats. Moreover, animal correlations of health and welfare with performance traits were mostly either non-significantly different from zero or favourable, implying that improving one would also benefit the other set of traits.

Few previous studies have estimated the repeatability of milk SCC, an indicator of subclinical mastitis, and significant estimates (0.31–0.59) have been reported in Saanen, Alpine, and mixed populations of goats [14,16,17,21]. Repeatability estimates of SCC reported in the present study for Eghoria and Skopelos goats were within the aforementioned range. The value found in Damascus goats was lower (0.22), which could be possibly attributed to differences in the studied breeds and/or farming systems and practices.

The ability of milk SCC to predict subclinical mastitis is reportedly lower in goats compared to dairy sheep and cows since it may be influenced by many more physiological and environmental factors in the former [36,37,38]. Nevertheless, there is no literature on the repeatability of other udder health indicator traits. The present study is the first to investigate TVC as well as combinations of SCC and TVC in this regard. The latter included a weighted SMI in which a greater emphasis (60%) was placed in SCC due to ease of measurement and cost-effectiveness of the trait [37], and three additional udder health phenotypes (UHP1, UHP2, and UHP3) defined according to thresholds for SCC and TVC (>10^3^ cells/mL and >2 × 10^4^ cfu/mL, respectively). These thresholds were based on the study by Gelasakis et al. [7] in which ca. 80% of the goat milk samples had exceeded both thresholds and found to have positive milk microbiological cultures. According to our results, significant between-animal variation exists for all the above udder health traits in Eghoria, Skopelos, and Damascus goats. UHP1 seems to be the best candidate since it was the most repeatable trait (0.43–0.59). However, it should be noted that including UHP1 in management and selection practices would require routine recording of TVC, hence implying an additional cost for the farmer. In this regard, decisions could be based only on SCC given that significant repeatability estimates were found for this trait in all three breeds. Moreover, despite previous research suggesting a lower association with intramammary infections in goats compared to other species, Rupp et al. [13] recently provided evidence of SCC being a valuable predictor of subclinical mastitis in Alpine goats that could be efficiently used for selection purposes and informed culling decisions.

Regarding endoparasites, the repeatability of GIN FEC has been widely studied in extensively reared dairy goats and significant medium to high estimates (0.25–0.84) have been reported [9,10,22] in cases of natural infection. These findings are in broad accordance with those obtained in the present study in Eghoria goats, although much lower estimates (0.11) are reported here. This could be associated with the small sample size (*n* = 84) and number of records (*n* = 584) available in our study. There are no previous reports on the repeatability of cestode FEC and lungworm FLC. In our study, significant estimates were found only for cestode FEC in Eghoria goats. However, cestode and lungworm load manifested in FEC and FLC was low in our data, which may have led to an underestimation of between-animal variation [20]. Therefore, in a separate series of analyses, infection with each endoparasite was defined as a binary trait (presence or absence of infection). These analyses revealed increased between-animal variation compared to the corresponding traits based on FEC and FLC. Higher repeatability estimates (026–0.32) were derived for GIN, lungworm, and overall endoparasite infection in Eghoria goats, which were within the range of previous reports [9,22]. Since data distribution in our study indicates that lungworm and cestode infections may not be a serious problem in the three studied dairy goat breeds, future research with a higher sample size and parasitic load could help to further investigate between-animal variation for these traits.

To our knowledge, there are no previous reports on the repeatability of infestation with ectoparasites in goats. In beef cattle, significant estimates (ca. 0.50) have been reported for myiasis resulting from buffalo and horn flies [39,40]. These findings are in accordance with those of the present study in Skopelos goats for myiasis with *Przhevalskiana silenus* larvae. Moreover, previous reports in cattle [41,42,43,44] and sheep [45,46] have indicated significant repeatability estimates for tick infestation (0.21–0.45 and 0.39–0.44, respectively) under natural or experimental challenge. However, other estimates were found to not significantly differ from zero in Angus cattle [47]. The latter results are in general agreement with those of our study in Eghoria and Skopelos goats. According to Giglioti et al. [44], animal age and frequency of examination and disease recording could influence the repeatability estimates of tick infestation. In the present study, animals of 2–5 years of age were included, and records were collected in one-month intervals. Further research within different age groups and with shorter examination intervals is warranted. Moreover, additional data with a higher frequency of ectoparasite infestation would be needed to properly estimate between-animal variance of these traits.

All studied welfare traits were significantly repeatable, except for those associated with problems in very low frequency (ocular discharge, diarrhoea, injuries, and hernias in all breeds, lameness and arthritis in Eghoria and Skopelos goats, and nasal discharge in Skopelos goats). According to available literature, our study is the first to evaluate the repeatability of welfare traits in goats. Moreover, there is a notable shortage of relevant literature in other farm animal species for most welfare-related traits. Currently, there are several genetic studies of lameness in dairy cows and significant heritability estimates (0.10–0.54) have been reported [48,49,50,51]. Heritability can indicate the lower limit of repeatability. In this regard, the moderate to high repeatability estimate of lameness found in Damascus goats is in general agreement with the results of the aforementioned studies, although the latter were on another species. Moderate to high repeatability estimates were also found for other studied limb problems (arthritis and overgrown claws) in the Damascus breed. Moderate estimates were found for udder problems with the most repeatable trait being udder asymmetry in all breeds. Udder asymmetry is often the result of a former intramammary infection and is usually irreversible [25], thus explaining its high repeatability estimate. The very high repeatability (>0.90) of ear and horn injuries could also be possibly attributed to records pertaining to the same injuries rather than new incidents. Therefore, further research on distinct incidents of injuries is warranted.

Overall, according to our results, between-animal variance accounts for a significant proportion of the total phenotypic variance of most of the studied traits in Eghoria, Skopelos, and Damascus goats. Such results suggest that there is potential to improve goat health and welfare through management and selection practices. Specifically, results indicate that it is possible to predict the future health and welfare status of individual animals and identify those most probable to (i) be repeatedly infected with GIN and, consequently, produce excretions responsible for pasture contamination in Eghoria breed, (ii) contribute to high incidence of myiasis in Skopelos breed, and (iii) be prone to udder health and welfare issues in all breeds. Based on the above, preventive and control strategies could be applied selectively to individual animals rather than in the whole herd. This may contribute to reducing anthelmintic and antibiotic resistance, while being cost-effective for the farmer. These findings are consistent with outcomes from previous studies on other dairy goat breeds [9,10,22]. Moreover, selection to reduce subclinical mastitis, GIN infections, and myiasis and improve the welfare status of animals could be applied based on goat records early in life, thereby enabling timely and informed culling decisions.

Furthermore, given that between-animal variation is partly genetic, the studied traits with significant repeatability could also be heritable. In our study, it was not possible to disentangle the additive genetic variance from the permanent environmental variance due to lack of pedigree data. Previous research in goats supports the possibility of selective breeding towards resistance to subclinical mastitis based on SCC [14,15,16,17] and resistance to GIN [11,18,19,52]. Myiasis resistance has also been shown to be moderately heritable in beef cattle (0.47) [39]. Finally, low but significant heritability estimates (≤0.15) have been generally reported for lameness in cattle and sheep [48,49,50,53] and for overall foot health in dairy cows [54].

According to the findings of the present study, implementing management and selection practices to improve goat health is not expected to compromise performance of the studied breeds. All animal correlations between udder health and milk production traits were either non-significant or favourable. Given that part of these correlations has a genetic basis, our findings are consistent with previous genetic correlations of SCC with milk, fat and protein yield reported in Alpine and Saanen goats [15]. However, Scholtens et al. [21] reported slightly positive and unfavourable correlations of SCC with milk and protein yield in New Zealand goats. Nevertheless, the generally favourable correlations between udder health and milk production traits suggest that it is possible to simultaneously improve udder health and milk production of the three studied breeds. Moreover, this is not expected to compromise goat BCS since all correlations with udder health traits did not significantly differ from zero.

In Eghoria goats, animal correlations of GIN infection with milk production traits were favourable, in agreement with the results of Morris et al. [23] in Saanen goats. However, unfavourable genetic correlations between these traits have been reported in Saanen goats by Heckendron et al. [11]. Contradictory results could be possibly explained by differences in GIN species, pasture infection pressure, and/or immunological responses of animals in the different studies [11,55]. Moreover, the present is the first study to estimate correlations between GIN FEC and BCS in goats. The non-significant estimates found in our study agree with the genetic correlations reported by Boareki et al. [56] in sheep. There is no literature regarding the association between myiasis and goat performance. In beef cattle, unfavourable genetic correlations have been reported of myasis with growth traits [40]. These findings were not supported by our study, where no significant animal correlations were found with BCS and milk production traits. The discrepancy may be attributed to species differences, as well as the type and severity of myiasis.

Our results suggest that the welfare status of the studied dairy goat breeds could also be improved without compromising their overall performance since, in most cases, no significant relevant associations were reported. Moreover, we found that skin lesions and udder asymmetry could be reduced by improving milk quality, based on the favourable animal correlations with some milk composition traits in Eghoria and Damascus goats, respectively. However, it should be noted that some welfare and milk production traits were unfavourably associated. Specifically, Skopelos goats with higher milk production and better milk quality seem to be more vulnerable to limb problems (mostly overgrown claws) and udder problems (mostly udder abscesses). Such results could be partly attributed to environmental factors such as grazing on soft and wet soil, inadequate flooring, and/or high supplementation with concentrate feedstuffs [5,25]. Nevertheless, between-animal covariance also includes genetic components. Relevant literature in goats is scarce. In dairy cows, an unfavourable genetic relationship between milk production and welfare indicators, including lameness and udder-type traits, has been reported [57]. Uribe et al. [54] also reported an antagonistic association between milk production traits and culling for limb problems. Such results are in agreement with the animal correlations of the present study for Skopelos goats. Therefore, selecting for higher milk production could possibly increase limb and udder issues in Skopelos goats. However, it might be possible to improve milk production while also reducing udder abscess through practices targeting minimisation of intramammary infections and udder injuries. Udder abscess usually results from intramammary infections and/or injuries, which as shown in the present study, are not unfavourably associated with milk production traits. In addition, such antagonistic traits can be combined in a suitably constructed phenotypic selection index towards overall improvement. This index would be based on adjusted animal phenotypes, each weighed according to relative economic importance, between-animal trait variance, and co-variance with the other traits of interest [58,59,60,61], and applied to inform culling decisions. Finally, results also suggest that arthritis and overall limb issues could be reduced in Damascus goats by improving BCS or maintaining it at desirable levels (score of 2.5 to 3). This finding is in accordance with previous research in dairy cows that reported favourable genetic correlations between foot health and BCS [51,62,63].

Future research aiming to estimate genetic correlations between the studied traits would provide results to underpin comprehensive selective breeding and genetic improvement programmes. However, this would require the collection of accurate pedigree data. As previously indicated, in low-input pastoral farming systems, pedigree recording is hindered by poor infrastructure, uncontrolled natural mating, and absence of artificial insemination [5,6,7,8,9,10,11,12,13,14,15,16,17,18,19,20]. Moreover, farmers are not well informed of the benefits of genetic improvement and reluctant to invest time and resources in record keeping [20]. However, there are possibilities to overcome some of these challenges through genomic evaluation and selection practices. Specifically, genotyping key animals with genome-wide DNA arrays could be a promising alternative to pedigree data availability in low-input dairy goat systems. Customised arrays for genomic markers identified to be associated with the studied traits could be developed for this purpose [20]. However, a complete feasibility and economic analysis would be needed to determine the optimal use of genomic technologies in low-input systems.

Finally, when practices to improve goat performance, health, and welfare are systematically implemented, it is advisable to periodically carry out welfare and performance assessments to assess improvements. Selection, even at the phenotypic level, could result in allelic frequency changes in the populations over time [64,65], which might impact on the estimation of (co)variance components. Moreover, ongoing changes in environmental conditions, including climate change, may also affect between-animal (co)variance estimates for many traits of interest [66].

## 5. Conclusions

Results of the present study indicate that there is significant between-animal variation for udder health, resistance to parasitism, and welfare traits to support management and selection practices aiming to improve the health and welfare status of Eghoria, Skopelos, and Damascus goats reared under low-input pastoral systems. Such practices could be implemented without compromising animal performance. Importantly, in most cases, subclinical mastitis, GIN infection, udder asymmetry, and skin lesions could be reduced when selecting for higher milk yield and quality. In some cases, arthritis and overall limb issues could be reduced by improving animal BCS. Few antagonistic trait associations revealed in our study, exemplified by the unfavourable correlation of certain welfare issues with milk production, could be addressed with appropriate management practices and possibly the implementation of a phenotypic selection index to underpin culling decisions. In all cases, accurate and systematic record keeping is essential for improving overall goat performance in low-input pastoral systems.

## Figures and Tables

**Table 1:** Descriptive statistics of milk production, body condition score, udder health (SCC, TVC, SMI) and parasitism (faecal egg and larval counts) traits in the studied goat breeds.

	Eghoria		Skopelos		Damascus	
Trait	N ^1^	Mean (±SD ^2^)	N ^1^	Mean (±SD ^2^)	N ^1^	Mean (±SD ^2^)
Daily milk yield (g)	2597	829.32 (379.64)	2766	1359.53 (738.40)	2124	1827.88 (1053.49)
Daily fat yield (g)	2561	39.46 (17.67)	2685	63.06 (31.85)	2110	76.49 (40.77)
Daily protein yield (g)	2563	30.82 (13.59)	2693	50.39 (26.69)	2095	65.00 (33.32)
Daily lactose yield (g)	2562	36.91 (17.96)	2688	59.77 (32.55)	2089	78.29 (45.47)
Daily SNF ^3^ yield (g)	2567	75.03 (34.65)	2695	122.40 (65.45)	2111	158.90 (87.48)
BCS ^4^ (1–5)	2920	2.31 (0.37)	2830	2.54 (0.32)	2298	2.44 (0.41)
SCC ^5^ (×10^3^ cells/mL)	2673	2050.43 (3590.82)	2167	1999.01 (1990.09)	2001	2816.54 (4125.64)
TVC ^6^ (×10^3^ cfu/mL)	2858	157.26 (415.80)	2657	97.6 (319.21)	1969	201.00 (463.86)
SMI ^7^	2667	−0.01 (0.91)	2087	0.00 (0.93)	1734	−0.04 (0.87)
GIN ^8^ FEC ^9^ (eggs/g)	584	267.29 (426.67)	546	8.52 (28.18)	814	93.30 (360.23)
Cestode FEC ^9^ (eggs/g)	584	1.80 (16.07)	546	5.59 (32.92)	814	0.98 (16.97)
Lungworm FLC ^10^ (larvae/g)	584	6.51 (33.01)	545	10.18 (59.19)	814	0.06 (1.75)

^1^ N = number of records; ^2^ SD = standard deviation; ^3^ SNF = solids-non-fat; ^4^ BCS = body condition score (1 = emaciated, 5 = obese); ^5^ SCC = somatic cell count; ^6^ TVC = total viable count; ^7^ SMI = subclinical mastitis index; ^8^ GIN = gastrointestinal nematodes; ^9^ FEC = faecal egg count; ^10^ FLC = faecal larval count.

**Table 2:** Frequency (%) of udder health phenotypes derived from milk somatic cell count and total viable count in the studied goat breeds.

Trait	Levels	Eghoria	Skopelos	Damascus
UHP1 ^1^	0	73.68	76.95	66.49
	1	26.32	23.05	33.51
UHP2 ^2^	0	50.36	54.86	40.60
	1	23.32	22.09	25.89
	2	26.32	23.05	33.51
UHP3 ^3^	0	50.36	54.86	40.60
	1	8.77	6.37	6.46
	2	14.55	15.72	19.43
	3	26.32	23.05	33.51

^1^ UHP1 = scored as 0 if somatic cell count ≤10^6^ cells/mL and/or total viable count ≤2 × 10^4^ cfu/mL, or 1 if somatic cell count >10^6^ cells/mL and total viable count >20 × 10^3^ cfu/mL; ^2^ UHP2 = scored as 0 if somatic cell count ≤10^6^ cells/mL and total viable count ≤2 × 10^4^ cfu/mL, 1 if somatic cell count >10^6^ cells/mL or total viable count >2 × 10^4^ cfu/mL, respectively, or 2 if somatic cell count >10^6^ cells/mL and total viable count >2 × 10^4^ cfu/mL; ^3^ UHP3 = scored as 0 if somatic cell count ≤10^6^ cells/mL and total viable count ≤2 × 10^4^ cfu/mL, 1 if only total viable count >2 × 10^4^ cfu/mL, 2 if only somatic cell count ≤10^6^ cells/mL or 3 if somatic cell count >10^6^ cells/mL and total viable count >2 × 10^4^ cfu/mL.

**Table 3:** Frequency (%) of parasitism in the studied goat breeds.

	Eghoria		Skopelos		Damascus	
Trait	N ^1^	Frequency (%)	N ^1^	Frequency (%)	N ^1^	Frequency (%)
Tick infestation	2921	13.63	2830	8.80	2298	0.26
Myiasis	2921	0.24	2830	4.55	2298	0.00
GIN ^2^ infection	584	66.44	546	11.72	814	27.40
Cestode infection	584	2.05	546	4.76	814	0.61
Lungworm infection	584	5.14	545	7.34	814	0.12
Endoparasite infection	584	70.03	545	19.23	814	27.64

^1^ N = number of records; ^2^ GIN = gastrointestinal nematodes.

**Table 4:** Frequency (%) of welfare issues in the studied goat breeds.

		Eghoria		Skopelos		Damascus	
Body Part	Trait	N ^1^	Frequency (%)	N ^1^	Frequency (%)	N ^1^	Frequency (%)
Head	Ear injuries	2920	8.29	2830	1.80	2298	4.09
	Horn injuries	2920	11.23	2830	8.90	2298	18.28
	Head skin lesions	2920	7.60	2830	2.37	2298	8.66
	Nasal discharge	2920	10.14	2830	0.88	2298	2.39
	Ocular discharge	2920	0.41	2830	0.07	2298	0.57
	Head problems	2920	31.43	2830	13.36	2298	31.38
Body	Abscess	2921	1.51	2830	13.39	2298	9.88
	Diarrhoea	2920	0.51	2830	0.04	2298	0.52
	Injury	2921	0.45	2830	0.25	2298	0.35
	Hernia	2921	0.00	2830	0.28	2298	0.00
	Body problems	2921	3.15	2830	14.42	2298	10.92
Legs	Lameness	2921	0.10	2830	0.04	2298	1.39
	Overgrown claws	2920	4.35	2830	4.38	2298	37.21
	Arthritis	2920	0.03	2830	0.11	2298	4.79
	Leg problems	2921	4.45	2830	4.52	2298	39.21
Udder	Udder asymmetry	2920	38.18	2830	20.99	2298	32.46
	Udder abscess	2920	28.08	2830	25.51	2298	29.77
	Udder skin lesions	2920	1.51	2830	1.10	2298	2.05
	Udder problems	2920	54.25	2830	39.01	2298	51.61
Total	Skin lesions	2920	8.80	2830	3.43	2298	10.40
	Injuries	2920	19.25	2830	10.71	2298	21.76

^1^ N = number of records.

**Table 5:** Repeatability estimates (standard error in parentheses) for health (udder health and parasitism) traits in Eghoria, Skopelos, and Damascus goats.

Trait	Eghoria	Skopelos	Damascus
SCC ^1^ (×10^3^ cells/mL)	0.45 (0.02) *	0.47 (0.02) *	0.22 (0.02) *
TVC ^2^ (×10^3^ cfu/mL)	0.22 (0.02) *	0.30 (0.02) *	0.15 (0.02) *
SMI ^3^	0.20 (0.02) *	0.28 (0.03) *	0.10 (0.02) *
UHP1 ^4^ (0–1)	0.50 (0.04) *	0.59 (0.04) *	0.43 (0.05) *
UHP2 ^5^ (0–2)	0.08 (0.01) *	0.09 (0.02) *	0.08 (0.01) *
UHP3 ^6^ (0–3)	0.14 (0.02) *	0.15 (0.02) *	0.14 (0.02) *
Tick infestation (0–1)	0.03 (0.02)	0.03 (0.01)	NE ^10^
Myiasis (0–1)	NE ^10^	0.34 (0.10) *	NE ^10^
GIN ^7^ FEC ^8^ (eggs/g, Tukey)	0.11 (0.04) *	0.001 (0.002)	0.001 (0.002)
Cestode FEC ^8^ (eggs/g, Tukey)	0.09 (0.03) *	0.04 (0.03)	NE ^10^
Lungworm FLC ^9^ (larvae/g, Tukey)	0.04 (0.02)	0.001 (0.002)	NE ^10^
GIN ^7^ infection (0–1)	0.26 (0.07) *	0.06 (0.04)	0.01 (0.02)
Cestode infection (0–1)	0.23 (0.19)	0.27 (0.15)	NE ^10^
Lungworm infection (0–1)	0.32 (0.11) *	0.03 (0.03)	NE ^10^
Endoparasite infection (0–1)	0.29 (0.07) *	0.04 (0.03)	0.002 (0.005)

^1^ SCC = somatic cell count; ^2^ TVC = total viable count; ^3^ SMI = subclinical mastitis index; ^4^ UHP1 = scored as 0 if somatic cell count ≤10^6^ cells/mL and/or total viable count ≤2 × 10^4^ cfu/mL, or 1 if somatic cell count >10^6^ cells/mL and total viable count >2 × 10^4^ cfu/mL; ^5^ UHP2 = scored as 0 if somatic cell count ≤10^6^ cells/mL and total viable count ≤2 × 10^4^ cfu/mL, 1 if somatic cell count >10^6^ cells/mL or total viable count >2 × 10^4^ cfu/mL, respectively, or 2 if somatic cell count >10^6^ cells/mL and total viable count >2 × 10^4^ cfu/mL; ^6^ UHP3 = scored as 0 if somatic cell count ≤10^6^ cells/mL and total viable count ≤2 × 10^4^ cfu/mL, 1 if only total viable count >2 × 10^4^ cfu/mL, 2 if only somatic cell count ≤10^6^ cells/mL or 3 if somatic cell count >10^6^ cells/mL and total viable count >2 × 10^4^ cfu/mL; ^7^ GIN = gastrointestinal nematodes; ^8^ FEC = faecal egg count; ^9^ FLC = faecal larval count; ^10^ NE = not estimable. * Indicates statistically significant repeatability estimates (*p* < 0.05).

**Table 6:** Repeatability estimates (standard error in parentheses) for welfare traits in Eghoria, Skopelos, and Damascus goats.

Trait	Eghoria	Skopelos	Damascus
Ear injuries (0–1)	0.95 (0.01) *	0.96 (0.02) *	0.91 (0.03) *
Horn injuries (0–1)	0.99 (0.004) *	0.97 (0.01) *	0.98 (0.01) *
Head skin lesions (0–1)	0.18 (0.04) *	0.23 (0.07) *	0.43 (0.06) *
Nasal discharge (0–1)	0.21 (0.04) *	NE ^1^	0.25 (0.07) *
Ocular discharge (0–1)	NE	NE ^1^	NE ^1^
Head problems (0–1)	0.61 (0.03) *	0.78 (0.03) *	0.74 (0.03) *
Abscess (0–1)	0.41 (0.07) *	0.49 (0.04) *	0.46 (0.05) *
Diarrhoea (0–1)	NE ^1^	NE ^1^	NE ^1^
Injury (0–1)	NE ^1^	NE ^1^	NE ^1^
Hernia (0–1)	NE ^1^	NE ^1^	NE ^1^
Body problems (0–1)	0.22 (0.05) *	0.45 (0.04) *	0.42 (0.05) *
Lameness (0–1)	NE ^1^	NE ^1^	0.51 (0.08) *
Overgrown claws (0–1)	0.17 (0.05) *	0.46 (0.07) *	0.21 (0.04) *
Arthritis (0–1)	NE ^1^	NE ^1^	0.87 (0.04) *
Limb problems (0–1)	0.16 (0.05) *	0.45 (0.07) *	0.20 (0.04) *
Udder asymmetry (0–1)	0.52 (0.03) *	0.52 (0.04) *	0.63 (0.03) *
Udder abscess (0–1)	0.37 (0.03) *	0.50 (0.03) *	0.39 (0.04) *
Udder skin lesions (0–1)	0.43 (0.08) *	0.54 (0.08) *	0.29 (0.08) *
Udder problems (0–1)	0.36 (0.03) *	0.41 (0.03) *	0.46 (0.03) *
Total skin lesions (0–1)	0.16 (0.04) *	0.19 (0.05) *	0.35 (0.05) *
Total injuries (0–1)	0.95 (0.01) *	0.95 (0.01) *	0.93 (0.01) *

^1^ NE = not estimable. * Indicates statistically significant repeatability estimates (*p* < 0.05).

**Table 7:** Statistically significant (*p* < 0.05) animal correlations (standard error in parentheses) between health and milk production traits in Eghoria, Skopelos, and Damascus goats.

		Milk Production Traits				
Breed	Health Traits	Milk Yield (g, ln)	Fat Yield (g, ln)	Protein Yield (g, ln)	Lactose Yield (g, ln)	SNF Yield (g, ln)
Eghoria	SCC ^1^ (cells/ mL, ln)	−0.15 (0.06)	−0.22 (0.06)	NS ^8^	−0.20 (0.06)	−0.15 (0.07)
	TVC ^2^ (cfu/ mL, ln)	−0.14 (0.07)	−0.20 (0.07)	NS ^8^	−0.19 (0.07)	−0.12 (0.07)
	SMI ^3^	−0.21 (0.07)	−0.26 (0.08)	NS ^8^	−0.26 (0.07)	−0.19 (0.07)
	UHP1 ^4^ (0–1)	−0.21 (0.07)	−0.26 (0.08)	NS ^8^	−0.28 (0.07)	−0.20 (0.07)
	UHP2 ^5^ (0–2)	−0.22 (0.08)	−0.31 (0.09)	NS ^8^	−0.30 (0.08)	−0.21 (0.09)
	UHP3 ^6^ (0–3)	−0.18 (0.07)	−0.27 (0.07)	NS ^8^	−0.25 (0.07)	−0.18 (0.07)
	GIN ^7^ infection (0–1)	−0.39 (0.17)	NS ^8^	NS ^8^	−0.39 (0.18)	−0.37 (0.17)
	Endoparasite infection (0–1)	−0.39 (0.17)	NS ^8^	NS ^8^	−0.39 (0.17)	−0.36 (0.18)
Skopelos	SCC ^1^ (cells/mL, ln)	−0.22 (0.06)	−0.29 (0.06)	−0.18 (0.06)	−0.27 (0.06)	−0.23 (0.06)
	TVC ^2^ (cfu/mL, ln)	−0.23 (0.06)	−0.30 (0.07)	−0.18 (0.07)	−0.28 (0.06)	−0.24 (0.06)
	SMI ^3^	−0.24 (0.07)	−0.33 (0.07)	−0.20 (0.07)	−0.29 (0.07)	−0.25 (0.07)
	UHP1 ^4^ (0–1)	−0.30 (0.07)	−0.39 (0.07)	−0.24 (0.07)	−0.35 (0.07)	−0.31 (0.07)
	UHP2 ^5^ (0–2)	−0.31 (0.09)	−0.39 (0.09)	−0.22 (0.09)	−0.37 (0.09)	−0.31 (0.09)
	UHP3 ^6^ (0–3)	−0.22 (0.07)	−0.30 (0.07)	−0.15 (0.07)	−0.28 (0.07)	−0.22 (0.07)
Damascus	SCC ^1^ (cells/mL, ln)	−0.25 (0.09)	−0.24 (0.09)	NS ^8^	−0.27 (0.09)	−0.20 (0.09)
	UHP1 ^4^ (0–1)	−0.25 (0.09)	−0.21 (0.10)	NS ^8^	−0.33 (0.10)	NS ^8^
	UHP2 ^5^ (0–2)	−0.23 (0.09)	−0.34 (0.09)	NS ^8^	−0.33 (0.09)	−0.22 (0.09)
	UHP3 ^6^ (0–3)	−0.20 (0.08)	−0.30 (0.08)	NS ^8^	−0.27 (0.08)	−0.19 (0.08)

^1^ SCC = somatic cell count; ^2^ TVC = total viable count; ^3^ SMI = subclinical mastitis index; ^4^ UHP1 = scored as 0 if somatic cell count ≤10^6^ cells/mL and/or total viable count ≤2 × 10^4^ cfu/mL, or 1 if somatic cell count >10^6^ cells/mL and total viable count >2 × 10^4^ cfu/mL; ^5^ UHP2 = scored as 0 if somatic cell count ≤10^6^ cells/mL and total viable count ≤2 × 10^4^ cfu/mL, 1 if somatic cell count >10^6^ cells/mL or total viable count >2 × 10^4^ cfu/mL, respectively, or 2 if somatic cell count >10^6^ cells/mL and total viable count >2 × 10^4^ cfu/mL; ^6^ UHP3 = scored as 0 if somatic cell count ≤10^6^ cells/mL and total viable count ≤2 × 10^4^ cfu/mL, 1 if only total viable count >2 × 10^4^ cfu/mL, 2 if only somatic cell count ≤10^6^ cells/mL or 3 if somatic cell count >10^6^ cells/mL and total viable count >2 × 10^4^ cfu/mL; ^7^ GIN = gastrointestinal nematodes; ^8^ NS = non-significant.

**Table 8:** Statistically significant (*p* < 0.05) animal correlations (standard error in parentheses) between welfare and performance traits in Eghoria, Skopelos, and Damascus goats.

		Performance Traits					
Breed	Health Traits	Milk Yield (g, ln)	Fat Yield (g, ln)	Protein Yield (g, ln)	Lactose Yield (g, ln)	SNF Yield (g, ln)	BCS ^1^ (1–5)
Eghoria	Udder abscess (0–1)	NS ^2^	NS ^2^	0.17 (0.07)	NS ^2^	NS ^2^	NS ^2^
	Head skin lesions (0–1)	NS ^2^	NS ^2^	−0.28 (0.12)	NS ^2^	NS ^2^	NS ^2^
	Total skin lesions (0–1)	NS ^2^	−0.27 (0.13)	NS ^2^	NS ^2^	NS ^2^	NS ^2^
Skopelos	Udder abscess (0–1)	0.23 (0.06)	NS ^2^	0.24 (0.07)	0.18 (0.07)	0.21 (0.07)	NS ^2^
	Udder problems (0–1)	NS ^2^	NS ^2^	0.15 (0.07)	NS ^2^	NS ^2^	NS ^2^
	Overgrown claws (0–1)	0.36 (0.10)	0.44 (0.10)	0.38 (0.10)	0.37 (0.09)	0.40 (0.10)	NS ^2^
	Limb problems (0–1)	0.34 (0.09)	0.45 (0.10)	0.37 (0.10)	0.36 (0.10)	0.39 (0.10)	NS ^2^
Damascus	Udder asymmetry (0–1)	−0.24 (0.08)	−0.24 (0.08)	−0.20 (0.08)	−0.23 (0.08)	−0.23 (0.08)	NS ^2^
	Arthritis (0–1)	NS ^2^	NS ^2^	NS ^2^	NS ^2^	NS ^2^	−0.22 (0.09)
	Limb problems (0–1)	NS ^2^	NS ^2^	NS ^2^	NS ^2^	NS ^2^	−0.28 (0.10)

^1^ BCS = body condition score (1 = emaciated, 5 = obese); ^2^ NS = non-significant.

## Data Availability

Data presented in this study are contained within the article and supplementary material (Dataset S1 and Supplement 1).

## Supplementary Materials

The following supporting information can be downloaded at: https://www.mdpi.com/article/10.3390/vetsci9060289/s1, Dataset S1: Total dataset of goat traits used for the analyses; Table S1: Repeatability estimates (standard error in parentheses) for performance traits in Eghoria, Skopelos, and Damascus goats; all estimates were statistically greater than zero (*p* < 0.05); Table S2: Animal correlations (standard error in parentheses) of health and welfare traits with performance traits in Eghoria goats; Table S3: Animal correlations (standard error in parentheses) of health and welfare traits with performance traits in Skopelos goats; Table S4: Animal correlations (standard error in parentheses) of health and welfare traits with performance traits in Damascus goats.
