# Supplementary material for: Repeatability of Health and Welfare Traits and Correlation with Performance Traits in Dairy Goats Reared under Low-Input Farming Systems
Source: Vet Sci. 2022 Jun 11;9(6):289. doi: 10.3390/vetsci9060289 (PMC9231351; doi:10.3390/vetsci9060289)
Supplement: Supplementary file 1 [file vetsci-09-00289-s001.zip › Supplement 1.pdf]

Repeatability of health and welfare traits and correlation with performance traits in dairy goats reared under low-input farming systems

Sotiria Vouraki, Athanasios I. Gelasakis, Vasileia Fotiadou, Georgios Banos, Georgios Arsenos

Supplement 1

Table S1. Repeatability estimates (standard error in parentheses) for performance traits in Eghoria, Skopelos and Damascus goats; all estimates were statistically greater than zero (P<0.05).

Trait	Eghoria	Skopelos	Damascus
Milk yield (g, ln)	0.35 (0.02)	0.47 (0.02)	0.21 (0.02)
Fat yield (g, ln)	0.26 (0.02)	0.35 (0.02)	0.20 (0.02)
Protein yield (g, ln)	0.34 (0.02)	0.41 (0.02)	0.23 (0.02)
Lactose yield (g, ln)	0.34 (0.02)	0.46 (0.02)	0.20 (0.02)
SNF yield (g, ln)	0.35 (0.02)	0.44 (0.02)	0.21 (0.02)
BCS (1-5)	0.41 (0.02)	0.37 (0.02)	0.52 (0.02)

SNF=solids-non-fat; BCS=body condition score (1=emaciated, 5=obese).

Table S2. Animal correlations (standard error in parentheses) of health and welfare traits with performance traits in Eghoria goats.

Health and welfare traits	Performance traits					
	Milk yield (g, ln)	Fat yield (g, ln)	Protein yield (g, ln)	Lactose yield (g, ln)	SNF yield (g, ln)	BCS (1-5)
SCC ($\times 10^3$ cells/ml)	-0.15 (0.06)*	-0.22 (0.06)*	-0.08 (0.07)	-0.20 (0.06)*	-0.15 (0.07)*	0.08 (0.06)
TVC ($\times 10^3$ cfu/ml)	-0.14 (0.07)*	-0.20 (0.07)*	-0.03 (0.07)	-0.19 (0.07)*	-0.12 (0.07)*	0.12 (0.07)
SMI	-0.21 (0.07)*	-0.26 (0.08)*	-0.11 (0.07)	-0.26 (0.07)*	-0.19 (0.07)*	0.12 (0.07)
UHP1 (0-1)	-0.21 (0.07)*	-0.26 (0.08)*	-0.08 (0.07)	-0.28 (0.07)*	-0.20 (0.07)*	0.07 (0.07)
UHP2 (0-2)	-0.22 (0.08)	-0.31 (0.09)	-0.09 (0.08)	-0.30 (0.08)	-0.21 (0.09)	0.12 (0.08)
UHP3 (0-3)	-0.18 (0.07)	-0.27 (0.07)	-0.09 (0.07)	-0.25 (0.07)	-0.18 (0.07)	0.08 (0.07)
GIN FEC (eggs/g, Tukey)	-0.34 (0.19)	-0.34 (0.20)	-0.22 (0.21)	-0.34 (0.20)	-0.29 (0.20)	-0.25 (0.16)
Cestode FEC (eggs/g, Tukey)	-0.06 (0.21)	-0.06 (0.23)	0.02 (0.23)	-0.09 (0.24)	-0.04 (0.22)	0.11 (0.18)
Lungworm FLC (larvae/g, Tukey)	0.00 (0.29)	0.20 (0.29)	0.09 (0.31)	-0.07 (0.30)	0.00 (0.30)	0.09 (0.25)
GIN infection (0-1)	-0.39 (0.17)*	-0.30 (0.17)	-0.29 (0.18)	-0.39 (0.18)*	-0.37 (0.17)*	-0.13 (0.16)
Cestode infection (0-1)	-0.05 (0.39)	-0.10 (0.38)	0.07 (0.39)	-0.12 (0.37)	-0.01 (0.39)	0.22 (0.32)
Lungworm infection (0-1)	-0.02 (0.24)	0.10 (0.26)	0.05 (0.25)	-0.10 (0.24)	-0.02 (0.24)	0.04 (0.20)
Endoparasite infection (0-1)	-0.39 (0.17)*	-0.27 (0.18)	-0.27 (0.18)	-0.39 (0.17)*	-0.36 (0.18)*	-0.11 (0.16)
Tick infestation (0-1)	0.22 (0.22)	-0.02 (0.24)	0.11 (0.24)	0.16 (0.23)	0.16 (0.23)	0.15 (0.23)
Myiasis (0-1)	NE	NE	NE	NE	NE	NE
Ear injuries (0-1)	-0.02 (0.09)	0.02 (0.10)	0.01 (0.09)	-0.01 (0.09)	-0.02 (0.09)	-0.14 (0.08)
Horn injuries (0-1)	-0.08 (0.09)	0.01 (0.09)	-0.10 (0.09)	-0.11 (0.09)	-0.09 (0.09)	-0.07 (0.08)
Head skin lesions (0-1)	-0.21 (0.12)	-0.25 (0.13)	-0.28 (0.12)*	-0.20 (0.12)	0.25 (0.13)	-0.21 (0.12)
Nasal discharge (0-1)	0.16 (0.10)	0.16 (0.10)	0.09 (0.10)	0.11 (0.10)	0.12 (0.11)	0.19 (0.10)
Ocular discharge (0-1)	NE	NE	NE	NE	NE	NE
Head problems (0-1)	-0.06 (0.06)	-0.01 (0.08)	-0.05 (0.07)	-0.07 (0.07)	-0.06 (0.07)	-0.16 (0.09)
Abscess (0-1)	-0.19 (0.13)	-0.29 (0.16)	-0.19 (0.13)	-0.17 (0.13)	-0.19 (0.12)	-0.14 (0.12)
Diarrhoea (0-1)	NE	NE	NE	NE	NE	NE

Injury (0-1)	NE	NE	NE	NE	NE	NE
Hernia (0-1)	NE	NE	NE	NE	NE	NE
Body problems (0-1)	-0.20 (0.13)	-0.23 (0.12)	-0.25 (0.12)	-0.21 (0.12)	-0.22 (0.12)	-0.18 (0.12)
Lameness (0-1)	NE	NE	NE	NE	NE	NE
Overgrown claws (0-1)	0.16 (0.15)	0.15 (0.15)	0.15 (0.15)	0.13 (0.14)	0.16 (0.15)	-0.06 (0.14)
Arthritis (0-1)	NE	NE	NE	NE	NE	NE
Leg problems (0-1)	0.17 (0.14)	0.19 (0.15)	0.15 (0.15)	0.16 (0.14)	0.18 (0.14)	-0.12 (0.14)
Udder asymmetry (0-1)	-0.10 (0.07)	-0.12 (0.07)	-0.05 (0.07)	-0.08 (0.07)	-0.07 (0.07)	0.14 (0.08)
Udder abscess (0-1)	0.11 (0.07)	-0.04 (0.08)	0.17 (0.07)*	0.10 (0.07)	0.13 (0.07)	-0.04 (0.07)
Udder skin lesions (0-1)	0.00 (0.13)	-0.14 (0.14)	0.06 (0.13)	-0.02 (014)	-0.00 (0.13)	0.09 (0.13)
Udder problems (0-1)	-0.00 (0.08)	-0.08 (0.08)	0.07 (0.08)	-0.00 (0.07)	0.04 (0.08)	0.11 (0.07)
Skin lesions (0-1)	-0.19 (0.12)	-0.27 (0.13)*	-0.23 (0.12)	-0.18 (0.12)	-0.23 (0.12)	-0.13 (0.12)
Injuries (0-1)	-0.06 (0.07)	0.00 (0.08)	-0.05 (0.07)	-0.07 (0.07)	-0.07 (0.07)	-0.13 (0.07)

SNF=solids-non-fat; BCS=body condition score (1=emaciated, 5=obese); SCC=somatic cell count; TVC=total viable count; SMI=subclinical mastitis index; UHP1= scored as 0 if somatic cell count $\leq 10^6$ cells/ml and/or total viable count $\leq 2\times10^4$ cfu/ml, or 1 if somatic cell count $>10^6$ cells/ml and total viable count $>20\times10^3$ cfu/ml; UHP2= scored as 0 if somatic cell count $\leq 10^6$ cells/ml and total viable count $\leq 2\times10^4$ cfu/ml, 1 if somatic cell count $>10^6$ cells/ml or total viable count $>2\times10^4$ cfu/ml, respectively, or 2 if somatic cell count $>10^6$ cells/ml and total viable count $>2\times10^4$ cfu/ml; UHP3= scored as 0 if somatic cell count $\leq 10^6$ cells/ml and total viable count $\leq 2\times10^4$ cfu/ml, 1 if only total viable count $>2\times10^4$ cfu/ml, 2 if only somatic cell count $\leq 10^6$ cells/ml or 3 if somatic cell count $>10^6$ cells/ml and total viable count $>2\times10^4$ cfu/ml; GIN=gastrointestinal nematodes; FEC=faecal egg counts; FLC=faecal larval counts; NE=not estimable

*Indicates statistically significant correlation estimates (P<0.05).

Table S3. Animal correlations (standard error in parentheses) of health and welfare traits with performance traits in Skopelos goats.

Health and welfare traits	Performance traits					
	Milk yield (g, ln)	Fat yield (g, ln)	Protein yield (g, ln)	Lactose yield (g, ln)	SNF yield (g, ln)	BCS (1-5)
SCC (×10^3 cells/ml)	-0.22 (0.06)*	-0.29 (0.06)*	-0.18 (0.06)*	-0.27 (0.06)*	-0.23 (0.06)*	0.09 (0.06)
TVC (×10^3 cfu/ml)	-0.23 (0.06)*	-0.30 (0.07)*	-0.18 (0.06)*	-0.28 (0.06)*	-0.24 (0.06)*	0.06 (0.07)
SMI	-0.24 (0.07)*	-0.33 (0.07)*	-0.20 (0.07)*	-0.29 (0.07)*	-0.25 (0.07)*	0.04 (0.07)
UHP1 (0-1)	-0.30 (0.07)*	-0.39 (0.07)*	-0.24 (0.07)*	-0.35 (0.07)*	-0.31 (0.07)*	0.06 (0.08)
UHP2 (0-2)	-0.31 (0.09)*	-0.39 (0.09)*	-0.22 (0.09)*	-0.37 (0.09)*	-0.31 (0.09)*	0.03 (0.09)
UHP3 (0-3)	-0.22 (0.07)*	-0.30 (0.07)*	-0.15 (0.07)*	-0.28 (0.07)*	-0.22 (0.07)*	0.08 (0.07)
GIN FEC (eggs/g, Tukey)	-0.36 (0.48)	-0.22 (0.58)	0.12 (0.48)	-0.14 (0.57)	-0.38 (0.45)	0.04 (0.58)
Cestode FEC (eggs/g, Tukey)	0.21 (0.28)	0.16 (0.33)	0.37 (0.28)	NE	NE	NE
Lungworm FLC (larvae/g, Tukey)	0.26 (0.42)	0.11 (0.46)	0.06 (0.67)	0.08 (0.50)	0.08 (0.58)	0.04 (0.48)
GIN infection (0-1)	-0.03 (0.35)	-0.21 (0.34)	-0.06 (0.35)	-0.04 (0.35)	-0.05 (0.34)	-0.43 (0.23)
Cestode infection (0-1)	0.20 (0.24)	0.09 (0.24)	0.24 (0.23)	0.18 (0.24)	0.21 (0.24)	0.23 (0.23)
Lungworm infection (0-1)	-0.11 (0.45)	-0.12 (0.44)	-0.09 (0.44)	-0.10 (0.45)	-0.10 (0.46)	0.13 (0.44)
Endoparasite infection (0-1)	0.13 (0.36)	-0.08 (0.36)	0.15 (0.36)	0.14 (0.36)	0.12 (0.36)	-0.23 (0.35)
Tick infestation (0-1)	0.01 (0.24)	0.04 (0.24)	-0.05 (0.25)	-0.00 (0.25)	-0.02 (0.30)	0.04 (0.25)
Myiasis (0-1)	-0.22 (0.14)	-0.17 (0.14)	-0.22 (0.15)	-0.23 (0.14)	-0.28 (0.18)	0.33 (0.18)
Ear injuries (0-1)	0.07 (0.11)	0.06 (0.12)	0.07 (0.12)	0.07 (0.11)	0.07 (0.11)	-0.03 (0.12)
Horn injuries (0-1)	0.12 (0.08)	0.10 (0.09)	0.10 (0.09)	0.10 (0.09)	-0.03 (0.09)	0.01 (0.10)
Head skin lesions (0-1)	-0.10 (0.14)	0.04 (0.15)	-0.13 (0.14)	-0.07 (0.15)	-0.12 (0.26)	-0.10 (0.15)
Nasal discharge (0-1)	NE	NE	NE	NE	NE	NE
Ocular discharge (0-1)	NE	NE	NE	NE	NE	NE
Head problems (0-1)	0.10 (0.07)	0.11 (0.07)	0.08 (0.07)	0.08 (0.07)	0.08 (0.07)	-0.02 (0.07)
Abscess (0-1)	0.12 (0.08)	0.16 (0.08)	0.13 (0.08)	0.13 (0.08)	0.13 (0.08)	-0.05 (0.08)
Diarrhoea (0-1)	NE	NE	NE	NE	NE	NE

Injury (0-1)	NE	NE	NE	NE	NE	NE
Hernia (0-1)	NE	NE	NE	NE	NE	NE
Body problems (0-1)	0.11 (0.07)	0.14 (0.08)	0.10 (0.08)	0.10 (0.08)	0.10 (0.08)	-0.04 (0.08)
Lameness (0-1)	NE	NE	NE	NE	NE	NE
Overgrown claws (0-1)	0.36 (0.10)*	0.44 (0.10)*	0.38 (0.10)*	0.37 (0.09)*	0.40 (0.10)*	-0.12 (0.10)
Arthritis (0-1)	NE	NE	NE	NE	NE	NE
Leg problems (0-1)	0.34 (0.09)*	0.45 (0.10)*	0.37 (0.10)*	0.36 (0.10)*	0.39 (0.10)	-0.15 (0.10)
Udder asymmetry (0-1)	-0.08 (0.07)	-0.12 (0.07)	-0.04 (0.07)	-0.09 (0.07)	-0.08 (0.07)	0.13 (0.07)
Udder abscess (0-1)	0.23 (0.06)*	0.10 (0.07)	0.24 (0.07)*	0.18 (0.07)*	0.21 (0.07)*	0.09 (0.07)
Udder skin lesions (0-1)	0.21 (0.12)	0.16 (0.12)	0.19 (0.13)	0.20 (0.12)	0.51 (0.26)	0.08 (0.12)
Udder problems (0-1)	0.13 (0.07)	0.01 (0.07)	0.15 (0.07)*	0.08 (0.07)	0.11 (0.07)	0.17 (0.09)
Skin lesions (0-1)	0.12 (0.13)	0.15 (0.14)	0.06 (0.14)	0.13 (0.13)	0.10 (0.13)	0.01 (0.14)
Injuries (0-1)	0.15 (0.08)	0.10 (0.08)	0.13 (0.08)	0.13 (0.08)	0.14 (0.07)	0.02 (0.08)

SNF=solids-non-fat; BCS=body condition score (1=emaciated, 5=obese); SCC=somatic cell count; TVC=total viable count; SMI=subclinical mastitis index; UHP1= scored as 0 if somatic cell count ≤10^6 cells/ml and/or total viable count ≤2×10^4 cfu/ml, or 1 if somatic cell count >10^6 cells/ml and total viable count >20×10^3 cfu/ml; UHP2= scored as 0 if somatic cell count ≤10^6 cells/ml and total viable count ≤2×10^4 cfu/ml, 1 if somatic cell count >10^6 cells/ml or total viable count >2×10^4 cfu/ml, respectively, or 2 if somatic cell count >10^6 cells/ml and total viable count >2×10^4 cfu/ml; UHP3= scored as 0 if somatic cell count ≤10^6 cells/ml and total viable count ≤2×10^4 cfu/ml, 1 if only total viable count >2×10^4 cfu/ml, 2 if only somatic cell count ≤10^6 cells/ml or 3 if somatic cell count >10^6 cells/ml and total viable count >2×10^4 cfu/ml; GIN=gastrointestinal nematodes; FEC=faecal egg counts; FLC=faecal larval counts; NE=not estimable.

*Indicates statistically significant correlation estimates (P<0.05).

Table S4. Animal correlations (standard error in parentheses) of health and welfare traits with performance traits in Damascus goats.

**Health and welfare traits**	**Performance traits**					
	Milk yield (g, ln)	Fat yield (g, ln)	Protein yield (g, ln)	Lactose yield (g, ln)	SNF yield (g, ln)	BCS (1-5)
SCC (×10^3 cells/ml)	-0.25 (0.09)*	-0.24 (0.09)*	-0.08 (0.09)	-0.27 (0.09)*	-0.20 (0.09)*	-0.01 (0.07)
TVC (×10^3 cfu/ml)	-0.01 (0.10)	-0.04 (0.10)	0.11 (0.10)	-0.08 (0.10)	0.01 (0.10)	0.01 (0.09)
SMI	-0.16 (0.12)	-0.10 (0.12)	-0.02 (0.12)	-0.20 (0.12)	-0.12 (0.12)	0.08 (0.10)
UHP1 (0-1)	-0.25 (0.09)*	-0.21 (0.10)*	-0.04 (0.10)	-0.33 (0.10)*	-0.20 (0.10)	0.06 (0.09)
UHP2 (0-2)	-0.23 (0.09)*	-0.34 (0.09)*	-0.09 (0.09)	-0.33 (0.09)*	-0.22 (0.09)*	0.11 (0.08)
UHP3 (0-3)	-0.20 (0.08)*	-0.30 (0.08)*	-0.09 (0.08)	0.27 (0.08)*	0.19 (0.08)*	0.09 (0.07)
GIN FEC (eggs/g, Tukey)	0.10 (0.56)	0.16 (0.56)	0.08 (0.56)	0.07 (0.56)	0.08 (0.57)	-0.07 (0.58)
Cestode FEC (eggs/g, Tukey)	NE	NE	NE	NE	NE	NE
Lungworm FLC (larvae/g, Tukey)	NE	NE	NE	NE	NE	NE
GIN infection (0-1)	0.04 (0.57)	0.08 (0.58)	-0.01 (0.56)	-0.06 (0.56)	-0.02 (0.57)	0.04 (0.56)
Cestode infection (0-1)	NE	NE	NE	NE	NE	NE
Lungworm infection (0-1)	NE	NE	NE	NE	NE	NE
Endoparasite infection (0-1)	0.13 (0.55)	0.17 (0.57)	0.03 (0.57)	0.06 (0.57)	0.05 (0.57)	-0.02 (0.57)
Tick infestation (0-1)	NE	NE	NE	NE	NE	NE
Myiasis (0-1)	NE	NE	NE	NE	NE	NE
Ear injuries (0-1)	0.02 (0.12)	0.00 (0.13)	0.08 (0.12)	0.05 (0.13)	0.04 (0.12)	0.25 (0.13)
Horn injuries (0-1)	-0.05 (0.10)	-0.10 (0.10)	-0.09 (0.10)	-0.08 (0.10)	-0.07 (0.10)	-0.07 (0.08)
Head skin lesions (0-1)	0.15 (0.12)		0.19 (0.11)	0.16 (0.12)	0.17 (0.11)	0.01 (0.11)
Nasal discharge (0-1)	0.04 (0.17)	0.07 (0.18)	0.13 (0.17)	0.03 (0.17)	0.08 (0.18)	0.11 (0.15)
Ocular discharge (0-1)	NE	NE	NE	NE	NE	NE
Head problems (0-1)	0.02 (0.08)	0.02 (0.09)	0.04 (0.08)	0.02 (0.08)	0.03 (0.08)	0.05 (0.07)
Abscess (0-1)	0.08 (0.10)	0.12 (0.11)	0.13 (0.10)	0.13 (0.10)	0.11 (0.10)	-0.02 (0.08)

Diarrhoea (0-1)	NE	NE	NE	NE	NE	NE
Injury (0-1)	NE	NE	NE	NE	NE	NE
Hernia (0-1)	NE	NE	NE	NE	NE	NE
Body problems (0-1)	0.04 (0.10)	0.05 (0.11)	0.07 (0.10)	0.10 (0.11)	0.07 (0.10)	-0.02 (0.09)
Lameness (0-1)	0.12 (0.15)	0.19 (0.15)	0.20 (0.14)	0.15 (0.15)	0.17 (0.15)	-0.19 (0.11)
Overgrown claws (0-1)	-0.05 (0.12)	-0.20 (0.12)	-0.07 (0.13)	-0.06 (0.12)	-0.05 (0.12)	-0.17 (0.10)
Arthritis (0-1)	-0.05 (0.13)	-0.02 (0.12)	0.00 (0.12)	-0.05 (0.13)	-0.03 (0.12)	-0.22 (0.09)*
Leg problems (0-1)	-0.04 (0.12)	-0.16 (0.13)	-0.04 (0.12)	-0.04 (0.12)	0.01 (0.11)	-0.28 (0.10)*
Udder asymmetry (0-1)	-0.24 (0.08)*	-0.24 (0.08)*	-0.20 (0.08)*	-0.23 (0.08)*	-0.23 (0.08)*	0.01 (0.07)
Udder abscess (0-1)	0.10 (0.09)	-0.11 (0.09)	0.13 (0.09)	0.08 (0.09)	0.09 (0.09)	0.02 (0.08)
Udder skin lesions (0-1)	0.16 (0.16)	0.30 (0.15)	0.10 (0.17)	0.25 (0.17)	0.19 (0.16)	-0.02 (0.15)
Udder problems (0-1)	-0.11 (0.08)	-0.11(0.08)	-0.07 (0.08)	-0.11 (0.08)	-0.10 (0.09)	-0.00 (0.07)
Skin lesions (0-1)	0.19 (0.11)	0.20 (0.11)	0.22 (0.11)	0.22 (0.12)	0.21 (0.12)	0.00 (0.10)
Injuries (0-1)	0.01 (0.09)	-0.04 (0.09)	0.00 (0.09)	0.01 (0.09)	0.01 (0.09)	-0.00 (0.07)

SNF=solids-non-fat; BCS=body condition score (1=emaciated, 5=obese); SCC=somatic cell count; TVC=total viable count; SMI=subclinical mastitis index; UHP1= scored as 0 if somatic cell count $\leq 10^6$ cells/ml and/or total viable count $\leq 2\times10^4$ cfu/ml, or 1 if somatic cell count $>10^6$ cells/ml and total viable count $>20\times10^3$ cfu/ml; UHP2= scored as 0 if somatic cell count $\leq 10^6$ cells/ml and total viable count $\leq 2\times10^4$ cfu/ml, 1 if somatic cell count $>10^6$ cells/ml or total viable count $>2\times10^4$ cfu/ml, respectively, or 2 if somatic cell count $>10^6$ cells/ml and total viable count $>2\times10^4$ cfu/ml; UHP3= scored as 0 if somatic cell count $\leq 10^6$ cells/ml and total viable count $\leq 2\times10^4$ cfu/ml, 1 if only total viable count $>2\times10^4$ cfu/ml, 2 if only somatic cell count $\leq 10^6$ cells/ml or 3 if somatic cell count $>10^6$ cells/ml and total viable count $>2\times10^4$ cfu/ml; GIN=gastrointestinal nematodes; FEC=faecal egg counts; FLC=faecal larval counts; NE=not estimable.

*Indicates statistically significant correlation estimates ($P<0.05$).
